# Association of the oxidative balance score and chronic kidney disease: insights from the national health and nutrition examination survey 2009–2018

**DOI:** 10.3389/fnut.2024.1429191

**Published:** 2024-09-27

**Authors:** Xinyun Chen, Zheng Wu, Xingyu Hou, Wenhui Yu, Chang Gao, Shenju Gou, Ping Fu

**Affiliations:** ^1^Department of Nephrology, Kidney Research Institute, West China Hospital, Sichuan University, Chengdu, China; ^2^Business School, Sichuan University, Chengdu, China; ^3^School of Economics and Management, North China Electric Power University, Beijing, China

**Keywords:** oxidative balance score, chronic kidney disease, NHANES, antioxidants, cross-sectional study

## Abstract

**Introduction:**

Oxidative stress plays a pivotal role in the pathogenesis of chronic kidney disease (CKD). The oxidative balance score (OBS) was devised to quantify the overall oxidative state, integrating pro-oxidant and antioxidant influences from both dietary intake and lifestyle practices. The aim of this study was to delve into the relationship between the OBS and CKD within the adult population of the United States.

**Methods:**

Utilizing data from the National Health and Nutrition Examination Survey (NHANES) spanning 2009–2018, we derived the OBS from 16 dietary and four lifestyle factors. We employed weighted multivariate regression to probe the link between OBS and CKD. Additionally, we undertook subgroup analyses and applied Restricted Cubic Spline Regression (RCS) for further data analyses.

**Results:**

This study encompassed 19,444 participants. Logistic regression analysis consistently demonstrated a protective effect of higher OBS on CKD. In Model 3, each unit increase in OBS was associated with a 2% reduction in the risk of CKD (95% CI: 0.97–0.99, *p* < 0.001) and a 4% reduction in the risk of reduced estimated glomerular filtration rate (eGFR) (95% CI: 0.95–0.98, *p* < 0.001). The highest OBS quintile (Q4) also showed significant reductions in the risk of CKD (OR: 0.66, 95% CI: 0.53–0.82, *p* < 0.001) and reduced eGFR (OR: 0.51, 95% CI: 0.37–0.69, *p* < 0.001) in Model 3. RCS analysis revealed a linear relationship between OBS and CKD. Subgroup analyses indicated significant associations between OBS and CKD in most subgroups, except for those without hypertension or with cardiovascular disease. Additionally, interaction analyses demonstrated that age, hypertension, and diabetes significantly modify the association between OBS and CKD risk.

**Conclusion:**

An elevated OBS, reflecting a predominance of antioxidants, correlates with a diminished CKD risk in the American adult demographic. These insights emphasize the potential influence of oxidative equilibrium on the development of CKD.

## 1 Introduction

Chronic Kidney Disease (CKD) presents a formidable challenge to global health, characterized by persistent abnormalities in kidney structure or function persisting for over 3 months. Recent data delineated that the global incidence of CKD was approximately 9.1%, impacting an estimated 697.5 million individuals worldwide (1, 2).

CKD also accounts for approximately 1.2 million deaths annually, positioning it as the twelfth leading cause of mortality globally (1, 2). CKD is intricately linked with increased risks of cardiovascular complications, bone disorders, and afflictions in other tissues (3). Notably, impaired kidney function is attributable to 7.6% of cardiovascular disease fatalities (1, 2). Thus, the early detection and proactive management of CKD are paramount to curtailing its progression and minimizing its extensive.

The pathogenesis of CKD is governed by a multifaceted interplay of various elements, among which oxidative stress holds significant prominence. The term “oxidative stress,” coined by Helmut Sies, refers to an imbalance between the generation of oxidants and antioxidant defenses (4, 5). Superoxide (•O_2_), key reactive oxygen species (ROS) instigating oxidative stress, are chiefly produced through the activity of nicotinamide adenine dinucleotide phosphate (NADPH) oxidase in phagocytes and endothelial cells (6). The detoxification process is facilitated by superoxide dismutase (SOD), which converts superoxide into hydrogen peroxide (H_2_O_2_) (5). Pronounced dysregulation is observed in CKD, marked by increased NADPH oxidase activity and reduced SOD functionality, culminating in superoxide accumulation and subsequent oxidative stress (7). Elevations in oxidative-inflammatory stress markers were observed even at the nascent stages of CKD and escalate concomitantly with the progressive impairment of renal function (8–10). Indices related to oxidative stress, such as the triglyceride-glucose (TyG) index, also increase with the rising risk of CKD (11). This phenomenon May be ascribed to uremic toxins which have the capacity to promote ROS release, thereby exacerbating oxidative stress in CKD (12).

The Oxidative Balance Score (OBS) is a composite metric devised to evaluate the equilibrium between pro-oxidant and antioxidant exposures stemming from diet and lifestyle factors (13). In contrast to investigations concentrating solely on individual nutrients, the OBS offers a notable advantage by amalgamating diverse pro-oxidants and antioxidants inherent in diet and lifestyle, potentially rendering it a more precise indicator of overarching oxidative stress. A heightened OBS signifies augmented antioxidant exposure, thus conferring benefits in mitigating oxidative stress (14). Previous studies have substantiated that an elevated OBS correlates with a diminished risk of some diseases, including depression (13), kidney stone (14), cardiovascular disease (CVD) (15), periodontitis and others (16). Nonetheless, the relationship between OBS and CKD remains ambiguous. Therefore, a cross-sectional analysis was undertaken to scrutinize the correlation between OBS and CKD, leveraging data from the National Health and Nutrition Examination Survey (NHANES) spanning the years 2009 to 2018.

## 2 Materials and methods

### 2.1 Study population

**Figure 1:**
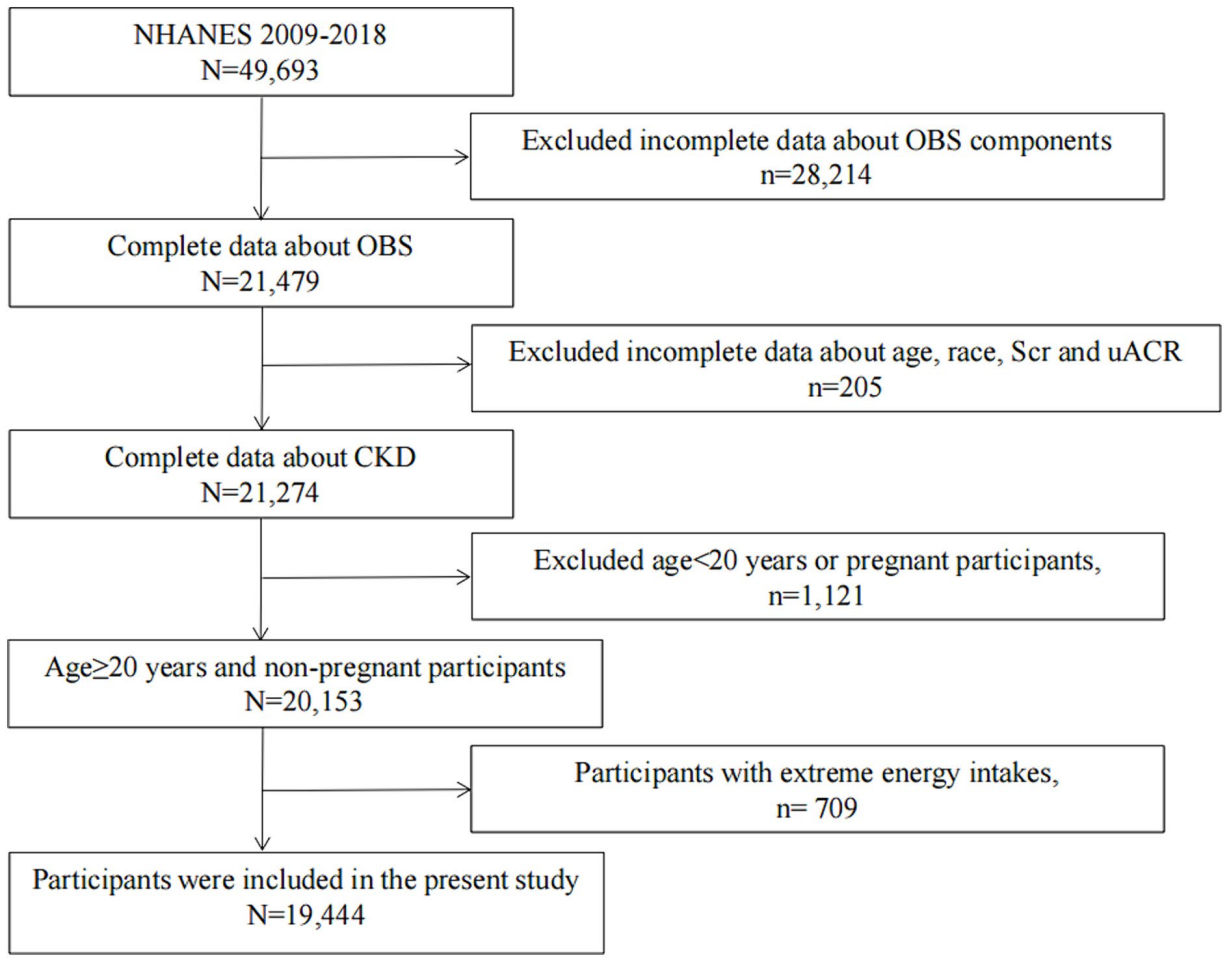
A flowchart showing the selection of study participants.

NHANES is an ongoing series of cross-sectional surveys approved and sponsored by the Centers for Disease Control and Prevention (CDC) to assess the health and nutritional status of the US population every 2 years.^1^ The NHANES underwent review and approval by the NCHS Research Ethics Review Board, with informed consent obtained from all participants. In this study, data from five NHANES cycles covering the period 2009–2018 were integrated, initially encompassing 49,693 participants. Subsequently, data on OBS components were unavailable for 28,214 participants. We excluded 205 participants due to missing data regarding age, race, serum creatinine, and urine albumin-to-creatinine ratio (UACR). Additionally, 1,121 participants under the age of 20 or pregnant were excluded. Then, individuals with unreliable energy intake (for males: <800 kcal/d or > 4,200 kcal/d, for females: <500 kcal/d or > 3,500 kcal/d) were excluded (*n* = 709). After applying these criteria, our final study population comprised 19,444 participants (Figure 1).

### 2.2 Data collection

#### 2.2.1 Exposure variable

The OBS is composed of 16 dietary and 4 lifestyle components, selected based on established associations between oxidative stress and various nutrients or lifestyle factors (14, 17). These components are categorized into 5 pro-oxidants (total fat, iron, alcohol intake, body mass index (BMI), and cotinine) and 15 antioxidants (including dietary fiber, carotene, vitamin B2, niacin, vitamin B6, total folate, vitamin B12, vitamin C, vitamin E, calcium, magnesium, zinc, copper, selenium, and physical activity).

The dietary components were selected based on their recognized roles as antioxidants, including vitamins C, E (*α*-tocopherol), B6, B12, *β*-carotene, and others (18). These antioxidants are included due to their ability to neutralize free radicals and reduce oxidative damage in the body. The OBS also includes dietary components known to have pro-oxidant effects, such as fatty acids and iron. These were selected because they can promote oxidative stress through processes like lipid peroxidation and iron-catalyzed oxidative reactions (18). Dietary and supplement data were collected from participants using two 24-h dietary recall interviews, encompassing both dietary and supplemental intake for calculating the dietary OBS. The recalls were conducted on non-consecutive days to capture variation in food consumption. Typically, one of these recalls was collected during a weekday, while the other was collected on a weekend to account for differences in dietary patterns between working days and weekends. The dietary recalls were conducted using a standardized protocol by trained interviewers who were proficient in dietary data collection techniques. These interviewers underwent rigorous training to ensure consistency and accuracy in data collection. The first 24-h recall was administered in person at the Mobile Examination Center (MEC), while the second recall was conducted via telephone 3 to 10 days later. For data analysis, the dietary intake data were processed and analyzed using the United States Department of Agriculture’s (USDA) Food and Nutrient Database for Dietary Studies (FNDDS). This database provides comprehensive information on the nutrient content of foods consumed in the United States and allows for accurate estimation of participants’ nutrient intake based on the foods reported in the 24-h recalls. The Automated Multiple-Pass Method (AMPM), developed by the USDA, was employed to enhance the accuracy of the dietary recalls by using a structured interview format that guides respondents through multiple passes of the recall to improve memory retrieval and reduce underreporting.

The lifestyle factors considered included physical activity, alcohol consumption, BMI, and cotinine levels. Physical Activity is included as it is known to enhance the body’s antioxidant defenses by activating cellular antioxidant signaling pathways (19). Regular exercise can increase the adaptive response to oxidative stress, thus contributing positively to the oxidative balance. Physical activity data were gathered via the Physical Activity Questionnaire (PAQ) as part of the NHANES, conducted in participants’ homes by trained interviewers using the Computer Assisted Personal Interview (CAPI) system. Physical activity levels were quantified as the product of the metabolic equivalent (MET) score, frequency of each activity per week, and duration of each activity. Based on this, physical activity was classified into three categories: low (<600 MET-minutes per week), moderate (600–3,000 MET-minutes per week), and high (>3,000 MET-minutes per week), which were assigned scores of 0, 1, and 2 points, respectively (20). Alcohol is included as a pro-oxidant lifestyle factor. It can increase the generation of ROS through the metabolism of ethanol to acetaldehyde, which can lead to oxidative damage (21). Alcohol consumption was assessed by calculating the average number of alcoholic drinks consumed per day on days when participants drank within the past 12 months. Alcohol intake was categorized into three groups: non-drinkers (<12 drinks/year), non-heavy drinkers (<1 drink/day for females and < 2 drinks/day for males), and heavy drinkers (≥1 drink/day for females and ≥ 2 drinks/day for males), with corresponding scores of 2, 1, and 0 points, respectively (17). Serum cotinine, a primary metabolite of nicotine with a significantly longer half-life, was used as an indicator of tobacco smoke exposure, covering both active and passive smoking. Smoking is included as a significant pro-oxidant factor because it disrupts the oxidative balance by reducing plasma antioxidant levels and increasing inflammatory markers, which leads to increased oxidative stress. BMI was calculated as weight in kilograms divided by height in meters squared (kg/m^2^). Measured through BMI, obesity is considered due to its association with oxidative stress via increased lipid peroxidation and systemic inflammation. All factors, except for alcohol consumption and physical activity were divided into gender-specific tertiles. Antioxidant factors were scored from 0 to 2 for the lowest to highest tertiles, whereas pro-oxidant factors were scored inversely, from 2 to 0. The total OBS score, which is the sum of all component scores, reflects a better oxidative balance with higher values. Table 1 details the classification and assigned scores for each component of the OBS.

**Table 1:** Oxidative balance score assignment scheme.

OBS components	Property	Male			Female		
		1	2	3	1	2	3
Dietary OBS							
Dietary fiber (g/d)^a^	A	<12.95	12.95–20.50	≥20.50	<11.25	11.25–17.25	≥17.25
Carotene (RE/d)^a^	A	<604.5	604.5–2175.2	≥2175.2	<684.0	684.0–2519.0	≥2519.0
Riboflavin (mg/d)^a^	A	<1.68	1.68–2.43	≥2.43	<1.34	1.34–1.90	≥1.90
Niacin (mg/d)^a^	A	<22.43	22.43–31.75	≥31.75	<16.15	16.15–22.86	≥22.86
Total folate (mcg/d)^a^	A	<320.0	320.0–480.5	≥480.5	<254.0	254.0–376.0	≥376.0
Calcium (mg/d)^a^	A	<729.5	729.5–1114.0	≥1114.0	<610.0	610.0–917.5	≥917.5
Zinc (mg/d)^a^	A	<9.36	9.36–13.72	≥13.72	<7.02	7.02–10.03	≥10.03
Magnesium (mg/d)^a^	A	<254.5	254.5–356.7	≥356.7	<207.5	207.5–287.0	≥287.0
Copper (mg/d)^a^	A	<0.99	0.99–1.41	≥1.41	<0.83	0.83–1.17	≥1.17
Selenium (mcg/d)^a^	A	<101.7	101.7–142.2	≥142.2	<74.1	74.1–105.7	≥105.7
Iron (mg/d)^a^	p	≥17.45	12.07–17.45	<12.07	≥13.57	9.43–13.57	<9.43
Total fat (g/d)^a^	P	≥98.24	67.26–98.24	<67.26	≥75.68	51.41–75.68	<51.41
Vitamin B6 (mg/d)^a^	A	<1.71	1.71–2.53	≥2.53	<1.28	1.28–1.88	≥1.88
Vitamin B12 (mcg/d)^a^	A	<3.56	3.56–6.66	≥6.66	<2.59	2.59–4.91	≥4.91
Vitamin C (mg/d)^a^	A	<38.20	38.20–95.80	≥95.80	<38.65	38.65–89.60	≥89.60
Vitamin E (ATE) (mg/d)^a^	A	<6.07	6.07–9.56	≥9.56	<5.15	5.15–8.09	≥8.09
Lifestyle OBS							
Physical activity (MET-minute/week)	A	<600	600–3,000	≥3,000	<600	600–3,000	≥3,000
Body mass index (kg/m2)	P	≥30.40	25.88–30.40	<25.88	≥31.86	25.60–31.86	<25.60
Alcohol	P	≥2 drinks/d	< 2 drinks/d	<12 drinks/year	≥1 drinks/d	< 1 drinks/d	<12 drinks/year
Cotinine (ng/mL)	P	≥0.97	0.02–0.97	<0.02	≥0.08	0.01–0.08	<0.01

OBS, oxidative balance score; A, antioxidant; P, prooxidant; RE, retinol equivalent; ATE, alpha-tocopherol equivalent; MET, metabolic equivalent. ^a^Total intake = dietary plus supplement intakes; inclusion of supplemental intake based on the availability of supplemental intake information.

#### 2.2.2 Outcome variable

CKD is characterized by a sustained abnormality in kidney structure or function lasting for 3 months or longer, indicated by either a reduced eGFR or albuminuria. A low eGFR was defined as an estimated glomerular filtration rate below 60 mL/min/1.73 m^2^, while albuminuria was identified as a UACR equal to or exceeding 30 mg/g. The eGFR was calculated using the Chronic Kidney Disease Epidemiology Collaboration study (CKD-EPI) equation based on Serum creatinine levels. Urinary albumin levels were measured using a solid-phase fluorescence immunoassay, and urinary creatinine levels were determined by employing an enzymatic method.

#### 2.2.3 Assessment of covariates and outcomes

The collection of covariate information primarily utilized three methods: surveys, physical assessments, and laboratory analyses. These surveys covered topics such as age, sex, ethnicity, educational attainment, marital status, income level, smoking habits, alcohol use, physical activity, caloric consumption, and a self-reported history of baseline health conditions, which included diabetes mellitus (DM), hypertension, and CVD. The physical assessments included measurements such BMI. Laboratory analyses covered a range of indicators including hemoglobin (HGB), albumin (ALB), total blood cholesterol (TC), blood lipid levels (TG), low-density and high-density lipoproteins (LDL and HDL, respectively), glycated hemoglobin (HbA1c), serum creatinine, blood urea nitrogen (BUN), and UACR.

Ethnic backgrounds were grouped into five categories: Mexican American, other Hispanic, Non-Hispanic White, Non-Hispanic Black, and others. Marital statuses were divided into six segments: married, widowed, divorced, separated, never married, and cohabitating. Educational attainments were classified into three levels: below high school, high school graduates, and above high school education. Household incomes were categorized based on the Federal Poverty Level (FPL) into ≤130% (reference category), >130–350, and > 350%.

CVD was identified in participants reporting a history of heart-related conditions like congestive heart failure, coronary artery disease, angina, or myocardial infarction. Hypertension was recognized in those with systolic pressure ≥ 130 mmHg or diastolic pressure ≥ 90 mmHg, a medical diagnosis of high blood pressure, or usage of blood pressure-lowering medications. DM diagnosis adhered to a comprehensive criterion that included a doctor’s diagnosis, use of diabetes medication or insulin, fasting blood sugar ≥126 mg/dL, 2 h-glucose levels ≥200 mg/dL during oral glucose tolerance test, or HbA1c ≥6.5%.

Smoking was categorized into never (less than 100 lifetime cigarettes), former (more than 100 lifetime cigarettes but currently non-smokers), and current smokers (more than 100 lifetime cigarettes and currently smoking). The mean daily calorie intake over a two-day period was determined through data collected from a food consumption survey. Detailed methodologies for these measurements can be found at https://www.cdc.gov/nchs/nhanes/.

### 2.3 Statistical analyses

Statistical analyses were conducted according to the Centers for Disease Control and Prevention guidelines for analysis of NHANES data. Given the complexity of the sampling method, the study employed a weighted statistical analysis approach. Baseline characteristics of the study population were categorized based on the quartiles of the OBS or the presence of CKD. Continuous metrics were presented as adjusted averages ± standard deviation or as the median alongside the interquartile span. Categorical variables were described using unweighted frequencies (weighted percentages).

To assess the link between OBS and CKD, four adjusted logistic regression models were developed. The trend significance was determined upon categorizing OBS into quartiles. The initial model, termed the Unadjusted Model, did not account for any confounding variables. The subsequent model, Model 1, incorporated adjustments for demographic details such as age, sex, and ethnicity. Model 2 expanded the adjustments to include lifestyle-related factors like drinking habits, smoking status, BMI, physical activity levels, and dietary caloric consumption. Model 3 made further adjustments for education levels, household income, and health history, specifically CVD, hypertension, and DM.

To validate the reliability of our results, we executed multiple sensitivity checks. We utilized a Restricted Cubic Spline (RCS) regression with three knots to probe the dose–response relationship between OBS and CKD. Four knots were selected and positioned specifically at the 5th, 35th, 65th, and 95th percentiles. To delve into the potential interactive influences of various covariates, we conducted subgroup and interaction analysis. These factors were pre-identified as possible modifiers of the effect. We included an interaction term in our analysis to assess the variation in relationships across different subgroups. All statistical procedures were carried out using the R software, version 4.2.2.^2^ A *p*-value of less than 0.05 was considered statistically significant.

## 3 Results

### 3.1 Participant characteristics

The analysis encompassed 19,444 participants, with a weighted mean age of 47.55, of whom 48% were males. The weighted baseline characteristics of the participants, segregated based on the presence or absence of CKD, were depicted in Table 2. It was observed that the group afflicted with CKD exhibited markedly lower values in OBS, inclusive of both dietary and lifestyle components. CKD group also displayed an elevated occurrence of hypertension, DM, CVD, and cancer. Table 3 delineates the fundamental demographic details and covariates of the study population, segmented according to quartiles of OBS. Individuals possessing higher quartiles of OBS were predominantly Non-Hispanic White, possessed greater levels of education, married, had higher Poverty Income Ratios (PIR), and consumed more energy. An inverse correlation was noted between OBS scores and the incidence of CKD. A significant reduction in CKD prevalence was observed from the lowest to the highest quartile of OBS scores (18.9% in Q1 to 10.6% in Q4, *p* < 0.001). This trend was accompanied by a decline in proteinuria prevalence (UACR ≥30 mg/g) and reduced eGFR (eGFR <60 mL/min/1.73 m^2^). Additionally, an increase in OBS scores was associated with a significant reduction in the occurrence of hypertension, DM, and CVD. Intriguingly, the rate of cancer incidence did not exhibit a correlation with OBS scores.

**Table 2:** Baseline characteristics of participants classified by CKD, weighted.

Characteristics^†^	Overall (*n* = 19,444)	Non-CKD (*n* = 16,138)	CKD (*n* = 3,306)	*p*-value
Age, years	47.55 ± 16.92	45.43 ± 15.93	60.66 ± 16.95	<0.001^*^
Male, *n* (%)	9,285 (48.0)	7,783 (48.9)	1,502 (42.0)	<0.001^*^
Race*, n* (%)				
Mexican American	2,854 (8.4)	2,447 (8.6)	407 (6.8)	
Other Hispanic	2,042 (5.9)	1,745 (6.1)	297 (4.9)	<0.001^*^
Non-Hispanic White	8,004 (67.6)	6,449 (67.1)	1,555 (70.5)	
Non-Hispanic Black	3,977 (10.2)	3,250 (10.0)	727 (11.4)	
Others	2,567 (7.9)	2,247 (8.2)	320 (6.4)	
Marital status, *n* (%)				
Married	9,943 (55.5)	8,293 (55.7)	1,650 (54.1)	
Widowed	1,397 (5.4)	822 (3.8)	575 (15.2)	
Divorced	2,122 (10.1)	1,692 (9.7)	430 (12.4)	<0.001^*^
Separated	624 (2.2)	512 (2.2)	112 (2.4)	
Never married	3,724 (18.5)	3,350 (19.8)	374 (10.3)	
Living with partner	1,634 (8.3)	1,469 (8.8)	165 (5.5)	
Education level, *n* (%)				
Less than high school	1,687 (4.3)	1,285 (3.9)	402 (6.7)	
High school	6,712 (31.3)	5,454 (30.6)	1,258 (36.0)	<0.001^*^
More than high school	11,045 (64.4)	9,399 (65.5)	1,646 (57.3)	
Household income, *n* (%)				
0–130% FPL	5,937 (20.5)	4,871 (20.2)	1,066 (22.6)	
>130–350% FPL	7,364 (35.6)	5,948 (34.5)	1,416 (42.1)	
>350% FPL	6,143 (43.9)	5,319 (45.3)	824 (35.3)	<0.001^*^
CVD, *n* (%)	1,481 (6.3)	810 (4.5)	671 (17.7)	<0.001^*^
Hypertension, *n* (%)	6,949 (31.8)	4,871 (27.3)	2,078 (59.4)	<0.001^*^
Diabetes, *n* (%)	3,568 (13.9)	2,233 (10.6)	1,335 (34.6)	<0.001^*^
Cancer, *n* (%)	1,842 (10.5)	1,238 (8.9)	604 (19.9)	<0.001^*^
BMI, kg/m^2^	29.17 ± 6.87	28.97 ± 6.76	30.40 ± 7.41	<0.001^*^
Smoking, *n* (%)				
No	11,373 (58.0)	9,571 (58.6)	1,802 (54.0)	
Former	4,391 (24.1)	3,411 (22.8)	980 (31.8)	<0.001^*^
Current	3,680 (17.9)	3,156 (18.5)	524 (14.2)	
Drinking, *n* (%)				
No	6,002 (23.9)	4,624 (22.1)	1,378 (35.4)	
Low-to-moderate	11,947 (66.9)	10,257 (68.7)	1,690 (55.7)	<0.001^*^
Heavy	1,495 (9.2)	1,257 (9.2)	238 (8.9)	
MET, *n* (%)				
<600 min/week	7,386 (33.5)	5,638 (31.0)	1,748 (48.4)	
600–3,999 min/week	7,106 (39.5)	6,111 (40.5)	995 (33.1)	
≥4,000 min/week	4,952 (27.1)	4,389 (28.5)	563 (18.5)	<0.001^*^
Calorie, kcal/day	1971 (1,528, 2,505)	2002 (1,558, 2,535)	1790 (1,382, 2,271)	<0.001^*^
OBS	20.80 ± 6.99	21.03 ± 6.98	19.37 ± 6.93	<0.001^*^
Dietary OBS	16.70 ± 6.67	16.89 ± 6.64	15.51 ± 6.73	<0.001^*^
Lifestyle OBS	4.10 ± 1.42	4.13 ± 1.42	3.87 ± 1.42	<0.001^*^
Hemoglobin, g/L	14.20 ± 1.43	14.27 ± 1.39	13.79 ± 1.62	<0.001^*^
Albumin, g/L	42.69 ± 3.35	42.87 ± 3.31	41.56 ± 3.40	<0.001^*^
TC, mmol/L	4.91 (4.24, 5.64)	4.91 (4.24, 5.61)	4.84 (4.16, 5.69)	0.314
TG, mmol/L	1.11 (0.76, 1.64)	1.07 (0.74, 1.60)	1.25 (0.88, 1.81)	<0.001^*^
HDL, mmol/L	1.40 ± 0.43	1.40 ± 0.42	1.38 ± 0.48	0.128
LDL, mmol/L	2.95 ± 0.90	2.96 ± 0.88	2.88 ± 0.98	0.002
HbA1c, %	5.63 ± 0.90	5.54 ± 0.75	6.17 ± 1.43	<0.001^*^
Serum creatinine, μmol/L	75.14 (63.65, 87.52)	73.37 (62.76, 84.86)	89.28 (69.84, 111.38)	<0.001^*^
Urea nitrogen, mmol/L	4.64 (3.57, 5.71)	4.64 (3.57, 5.71)	5.71 (4.28, 7.85)	<0.001^*^
eGFR,ml/min/1.73m^2^	94.19 ± 21.78	97.65 ± 18.14	72.82 ± 29.03	<0.001^*^
UACR, mg/g	6.58 (4.38, 11.57)	6.03 (4.18, 9.29)	41.25 (12.20, 93.08)	<0.001
Low eGFR, *n* (%)	1,600 (6.7)	–	1,600 (48.2)	<0.001^*^
Proteinuria, *n* (%)	2,248 (9.1)	–	2,248 (65.4)	<0.001^*^

FPL, family income to poverty; CVD, cardiovascular disease; BMI, body mass index; MET, metabolic equivalent; OBS, oxidative balance score; TC, total cholesterol; TG, triglyceride; eGFR, estimated glomerular filtration rate; UACR, urine albumin/creatinine ratio; CKD, chronic kidney disease. *p < 0.05, ^†^*n* (%): Unweighted numbers (weighted percentage).

**Table 3:** Baseline characteristics of participants classified by OBS quantiles, weighted.

Characteristics^†^	Overall (*n* = 19,444)	Classified by OBS quantiles				*p*- value
		Q1 (*n* = 4,959)	Q2 (*n* = 5,028)	Q3 (*n* = 5,189)	Q4 (*n* = 4,268)	
Age, years	47.55 ± 16.92	47.22 ± 17.47	48.09 ± 17.20	47.50 ± 16.77	47.37 ± 16.29	0.303
Male, *n* (%)	9,285 (48.0)	2,319 (45.3)	2,438 (48.9)	2,486 (48.2)	2,042 (49.1)	0.021
Race*, n* (%)						
Mexican American	2,854 (8.4)	651 (8.0)	735 (8.3)	799 (8.5)	669 (8.6)	
Other Hispanic	2,042 (5.9)	514 (6.3)	543 (6.1)	555 (5.6)	430 (5.6)	<0.001^*^
Non-Hispanic White	8,004 (67.6)	1,837 (61.9)	2,065 (66.6)	2,209 (69.5)	1,893 (71.3)	
Non-Hispanic Black	3,977 (10.2)	1,467 (16.7)	1,041 (11.0)	890 (8.2)	579 (6.1)	
Others	2,567 (7.9)	490 (7.0)	644 (8.0)	736 (8.2)	697 (8.4)	
Marital status, *n* (%)						
Married	9,943 (55.5)	2,177 (46.8)	2,536 (54.7)	2,810 (57.7)	2,420 (61.3)	
Widowed	1,397 (5.4)	414 (6.8)	389 (5.6)	357 (5.4)	237 (4.0)	
Divorced	2,122 (10.1)	626 (11.4)	609 (11.5)	496 (9.3)	391 (8.5)	<0.001^*^
Separated	624 (2.2)	215 (3.5)	147 (1.9)	164 (2.2)	98 (1.5)	
Never married	3,724 (18.5)	1,052 (21.6)	922 (17.3)	943 (17.5)	807 (17.9)	
Living with partner	1,634 (8.3)	475 (9.8)	425 (8.9)	419 (8.0)	315 (6.9)	
Education level, *n* (%)						
Less than high school	1,687 (4.3)	524 (5.9)	495 (4.9)	416 (3.6)	252 (3.0)	
High school	6,712 (31.3)	2,194 (42.8)	1,837 (35.0)	1,616 (28.9)	1,065 (20.4)	<0.001^*^
More than high school	11,045 (64.4)	2,241 (51.3)	2,696 (60.1)	3,157 (67.5)	2,951 (76.6)	
Household income, *n* (%)						
0–130% FPL	5,937 (20.5)	1,949 (29.9)	1,622 (22.3)	1,395 (17.0)	971 (14.4)	
>130–350% FPL	7,364 (35.6)	1,967 (40.3)	1,934 (36.7)	1,944 (35.1)	1,519 (30.8)	<0.001^*^
>350% FPL	6,143 (43.9)	1,043 (29.8)	1,472 (40.9)	1,850 (48.0)	1,778 (54.7)	
CVD, *n* (%)	1,481 (6.3)	519 (8.6)	404 (6.6)	354 (5.8)	204 (4.5)	<0.001^*^
Hypertension, *n* (%)	6,949 (31.8)	2,054 (36.7)	1,904 (34.8)	1,731 (29.9)	1,260 (26.6)	<0.001^*^
Diabetes, *n* (%)	3,568 (13.9)	1,123 (17.7)	1,007 (15.3)	884 (13.4)	554 (9.9)	<0.001^*^
Cancer, *n* (%)	1,842 (10.5)	492 (10.9)	491 (10.8)	457 (10.1)	402 (10.2)	0.762
BMI, kg/m^2^	29.17 ± 6.87	30.38 ± 7.20	29.58 ± 6.87	29.17 ± 6.77	27.68 ± 6.41	<0.001^*^
Smoking, *n* (%)						
No	11,373 (58.0)	2,498 (49.3)	2,872 (56.0)	3,171 (59.9)	2,832 (65.5)	
Former	4,391 (24.1)	1,038 (21.1)	1,161 (24.2)	1,204 (25.6)	988 (24.8)	<0.001^*^
Current	3,680 (17.9)	1,423 (29.6)	995 (19.8)	814 (14.5)	448 (9.7)	
Drinking, *n* (%)						
No	6,002 (23.9)	1,588 (26.1)	1,556 (24.3)	1,564 (23.0)	1,294 (22.7)	
Low-to-moderate	11,947 (66.9)	2,939 (63.5)	3,053 (66.1)	3,203 (66.7)	2,752 (71.0)	<0.001^*^
Heavy	1,495 (9.2)	432 (10.4)	419 (9.6)	422 (10.3)	222 (6.2)	
MET, min/week						
<600	7,386 (33.5)	2,430 (44.2)	2,037 (36.4)	1,818 (31.7)	1,101 (23.0)	
600–3,999	7,106 (39.5)	1,469 (32.0)	1,769 (37.7)	2,015 (41.2)	1,853 (45.9)	<0.001^*^
≥4,000	4,952 (27.1)	1,060 (23.8)	1,222 (25.9)	1,356 (27.1)	1,314 (31.1)	
Calorie, kcal/day	1,971 (1,528, 2,505)	1,425 (1,121, 1792)	1,812 (1,473, 2,227)	2,134 (1740, 2,610)	2,499 (2019, 3,034)	<0.001^*^
OBS	20.80 ± 6.99	10.95 ± 2.30	17.59 ± 1.70	23.49 ± 1.70	29.60 ± 2.11	<0.001^*^
Dietary OBS	16.70 ± 6.67	7.43 ± 2.27	13.64 ± 2.16	19.35 ± 2.10	24.91 ± 2.03	<0.001^*^
Lifestyle OBS	4.10 ± 1.42	3.53 ± 1.35	3.95 ± 1.41	4.14 ± 1.38	4.70 ± 1.29	<0.001^*^
Hemoglobin, g/L	14.20 ± 1.43	14.09 ± 1.56	14.25 ± 1.47	14.22 ± 1.38	14.23 ± 1.33	0.005
Albumin, g/L	42.6 ± 3.35	42.06 ± 3.52	42.61 ± 3.37	42.80 ± 3.26	43.21 ± 3.19	<0.001^*^
TC, mmol/L	4.91 (4.24, 5.64)	4.89 (4.19, 5.64)	4.94 (4.27, 5.66)	4.91 (4.24, 5.61)	4.89 (4.24, 5.59)	0.232
TG, mmol/L	1.11 (0.76, 1.64)	1.14 (0.79, 1.71)	1.13 (0.79, 1.69)	1.11 (0.76, 1.65)	1.02 (0.70, 1.51)	<0.001^*^
HDL, mmol/L	1.40 ± 0.43	1.35 ± 0.44	1.37 ± 0.43	1.41 ± 0.42	1.45 ± 0.43	<0.001^*^
LDL, mmol/L	2.95 ± 0.90	2.95 ± 0.92	2.97 ± 0.91	2.95 ± 0.87	2.94 ± 0.88	0.469
HbA1c, %	5.63 ± 0.90	5.71 ± 1.01	5.66 ± 0.95	5.62 ± 0.83	5.54 ± 0.82	<0.001^*^
Serum creatinine, μmol/L	75.14 (63.65, 87.52)	75.14 (63.65, 88.40)	75.14 (63.65, 89.28)	74.26 (62.76, 87.52)	75.14 (63.65, 85.75)	0.004
Urea nitrogen, mmol/L	4.64 (3.57, 5.71)	4.28 (3.57, 5.36)	4.64 (3.57, 5.71)	4.64 (3.93, 5.71)	5.00 (3.93, 6.07)	<0.001^*^
eGFR,ml/min/1.73m^2^	94.19 ± 21.78	94.15 ± 24.05	93.30 ± 22.11	94.61 ± 21.44	94.63 ± 19.61	0.096
UACR, mg/g	6.58 (4.38, 11.57)	7.32 (4.66, 13.89)	6.60 (4.30, 11.83)	6.52 (4.39, 10.87)	6.18 (4.18, 10.66)	<0.001^*^
CKD, *n* (%)	3,306 (13.9)	1,075 (18.2)	934 (15.4)	760 (12.3)	537 (10.6)	<0.001^*^
Low eGFR, *n* (%)	1,600 (6.7)	535 (8.7)	462 (8.1)	369 (6.1)	234 (4.3)	<0.001^*^
Proteinuria, *n* (%)	2,248 (9.1)	741 (12.4)	629 (9.5)	507 (7.8)	371 (7.3)	<0.001^*^

FPL, family income to poverty; CVD, cardiovascular disease; BMI, body mass index; MET, metabolic equivalent; OBS, oxidative balance score; TC, total cholesterol; TG, triglyceride; eGFR, estimated glomerular filtration rate; UACR, urine albumin/creatinine ratio; CKD, chronic kidney disease. *p < 0.05, ^†^*n* (%): Unweighted numbers (weighted percentage).

### 3.2 Relationship between OBS and kidney function

In this analysis, four stratified logistic regression models were utilized to examine the relationship between OBS and renal functionality, encompassing CKD susceptibility, diminished eGFR, and proteinuria occurrences, as delineated in Table 4. A consistent protective effect of elevated OBS levels on renal health was discerned across all models. In Model 3, subsequent to adjustments for demographic variables, lifestyle considerations, and medical histories, it was found that with every incremental unit of OBS, the likelihood of CKD was reduced by 2% (95% CI: 0.97–0.99, *p* < 0.001), the probability of reduced eGFR contracted by 4% (95% CI: 0.95–0.98, *p* < 0.001). However, for proteinuria, the continuous score has an OR of 0.99 (95% CI: 0.98–1.01, *p* = 0.098), which is not statistically significant. The highest quintile (Q4) of the OBS, significant reductions in risk are observed for CKD (OR: 0.66, 95% CI: 0.53–0.82, *p* < 0.001) and reduced eGFR (OR: 0.51, 95% CI: 0.37–0.69, *p* < 0.001), while for proteinuria, the OR is 0.78 (95% CI: 0.61–1.01, *p* = 0.053), which is not statistically significant. The *p* for trend across quintiles is significant for all outcomes indicating a general trend of decreasing odds with higher oxidative balance scores. Furthermore, the linkages between dietary and CKD risk were explored (Table 5), revealing a consistent inverse association across all models (all *p*-values <0.05). In contrast, the lifestyle OBS in Model 3 does not show a statistically significant association, with an OR of 0.95 (95% CI: 0.89–1.01, *p* = 0.115).

**Table 4:** Logistic regression analysis for the associations between OBS and kidney function, weighted.

	Unadjusted Model		Model 1		Model 2		Model 3	
	*OR* (95% CI)	*p*-value	*OR* (95% CI)	*p*-value	*OR* (95% CI)	*p*-value	*OR* (95% CI)	*p*-value
CKD								
Continuous	0.97 (0.96–0.97)	<0.001^*^	0.97 (0.96–0.97)	<0.001^*^	0.97 (0.96–0.98)	<0.001^*^	0.98 (0.97–0.99)	<0.001^*^
Q1	References		References		References		References	
Q2	0.82 (0.70–0.95)	0.009^*^	0.77 (0.65–0.90)	0.002^*^	0.79 (0.67–0.94)	0.006^*^	0.85 (0.71–1.01)	0.072
Q3	0.63 (0.55–0.72)	<0.001^*^	0.60 (0.52–0.69)	<0.001^*^	0.64 (0.53–0.76)	<0.001^*^	0.71 (0.58–0.86)	<0.001^*^
Q4	0.53 (0.46–0.62)	<0.001^*^	0.51 (0.44–0.60)	<0.001^*^	0.57 (0.47–0.69)	<0.001^*^	0.66 (0.53–0.82)	<0.001^*^
*P* for trend		<0.001^*^		<0.001^*^		<0.001^*^		<0.001^*^
eGFR<60 mL/min/1.73 m^2^								
Continuous	0.96 (0.95–0.97)	<0.001^*^	0.96 (0.95–0.97)	<0.001^*^	0.97 (0.95–0.98)	<0.001^*^	0.96 (0.95, 098)	<0.001^*^
Q1	References		References		References		References	
Q2	0.93 (0.76–1.13)	0.461	0.86 (0.68–1.09)	0.217	0.90 (0.70–1.14)	0.383	0.90 (0.70–1.16)	0.425
Q3	0.69 (0.56–0.83)	<0.001^*^	0.67 (0.54–0.83)	<0.001^*^	0.71 (0.55–0.91)	0.007^*^	0.71 (0.55–0.92)	0.010^*^
Q4	0.47 (0.38–0.59)	<0.001^*^	0.46 (0.36–0.59)	<0.001^*^	0.51 (0.38–0.69)	<0.001^*^	0.51 (0.37–0.69)	<0.001^*^
*P* for trend		<0.001^*^		<0.001^*^		<0.001^*^		<0.001^*^
UACR ≥30 mg/g								
Continuous	0.97 (0.96–0.98)	<0.001^*^	0.97 (0.96–0.98)	<0.001^*^	0.98 (0.96–0.99)	<0.001^*^	0.99 (0.98–1.01)	0.098
Q1	References		References		References		References	
Q2	0.74 (0.63–0.87)	<0.001^*^	0.72 (0.61–0.85)	<0.001^*^	0.76 (0.64–0.90)	0.002^*^	0.82 (0.69–0.99)	0.036^*^
Q3	0.60 (0.51–0.70)	<0.001^*^	0.59 (0.50–0.70)	<0.001^*^	0.64 (0.52–0.80)	<0.001^*^	0.74 (0.58–0.93)	0.010^*^
Q4	0.56 (0.47–0.67)	<0.001^*^	0.56 (0.46–0.67)	<0.001^*^	0.64 (0.51–0.81)	<0.001^*^	0.78 (0.61–1.01)	0.053
*P* for trend		<0.001^*^		<0.001^*^		<0.001^*^		0.035^*^

CKD, chronic kidney disease; eGFR, estimated glomerular filtration rate; UACR, urine albumin/creatinine ratio. *p < 0.05; OR: weighted odds ratio, Model 1 was adjusted for age + sex + race, Model 2 was adjusted for Model 1 + BMI + smoking status + drinking status + Physical activity + Calorie intake, and Model 3 was adjusted for Model 2 + CVD + hypertension + diabetes + education levels + household income.

**Table 5:** Logistic regression analysis for the associations between dietary/lifestyle OBS and CKD, weighted.

	*OR* (95% CI)	*p*-value
Dietary OBS		
Unadjusted Model	0.97 (0.96–0.98)	<0.001^*^
Model 1	0.97 (0.96–0.98)	<0.001^*^
Model 2	0.97 (0.96–0.98)	<0.001^*^
Model 3	0.98 (0.97–0.99)	<0.001^*^
Lifestyle OBS		
Unadjusted Model	0.88 (0.85–0.91)	<0.001^*^
Model 1	0.87 (0.84–0.91)	<0.001^*^
Model 2	0.89 (0.84–0.95)	<0.001^*^
Model 3	0.95 (0.89–1.01)	0.115

CKD, chronic kidney disease; eGFR, estimated glomerular filtration rate; OBS, oxidative balance score; UACR, urine albumin/creatinine ratio. *p < 0.05; OR: weighted odds ratio, Model 1 was adjusted for age + sex + race, Model 2 was adjusted for Model 1 + BMI + smoking status + drinking status + Physical activity + Calorie intake, and Model 3 was adjusted for Model 2 + CVD + hypertension + diabetes + education levels + household income.

### 3.3 The dose–response association between OBS and kidney function

**Figure 2:**
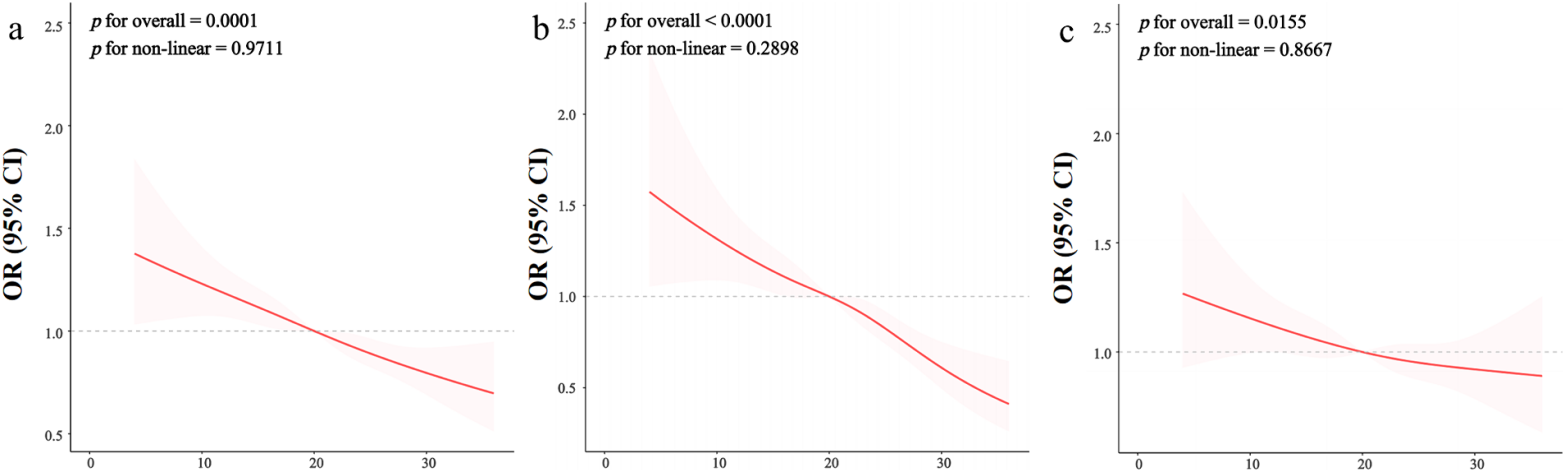
The restricted cubic spline regression between OBS and kidney function. The dose–response relationship was assessed using restricted cubic spline regression for the OBS in relation to kidney function outcomes, including chronic kidney disease (CKD) **(A)**, decreased estimated glomerular filtration rate (eGFR) **(B)**, and proteinuria prevalence **(C)**, with adjustments for covariates as specified in Model 3. The red line represents the odds ratio (OR), while the shaded pink area indicates the 95% confidence interval (CI). Decreased eGFR is defined as an eGFR less than 60 mL/min/1.73 m^2^, and proteinuria prevalence is defined as a urine albumin-to-creatinine ratio (UACR) of 30 mg/g or greater. The linearity test results suggest that the relationship between OBS and each kidney function measure is non-linear, indicating potential thresholds or varying effects at different OBS levels.

To investigate the dose–response relationship between OBS and the prevalence of CKD, the analysis was conducted using Restricted Cubic Splines (RCS) regression. Figure 2A illustrated that the relationship between OBS levels and CKD prevalence appeared to be linear, as indicated by *p* for overall equal to 0.0001 and *p* for non-linear equal to 0.9711. Furthermore, a linear correlation was observed between continuous OBS values and reduced eGFR, as depicted in Figure 2B (*p* for overall <0.0001, *p* for non-linear = 0.2898). Similarly, Figure 2C demonstrated a linear correlation between continuous OBS measures and the occurrence of proteinuria (*p* for overall = 0.0155, *p* for non-linear = 0.8667).

### 3.4 Subgroup analysis

**Figure 3:**
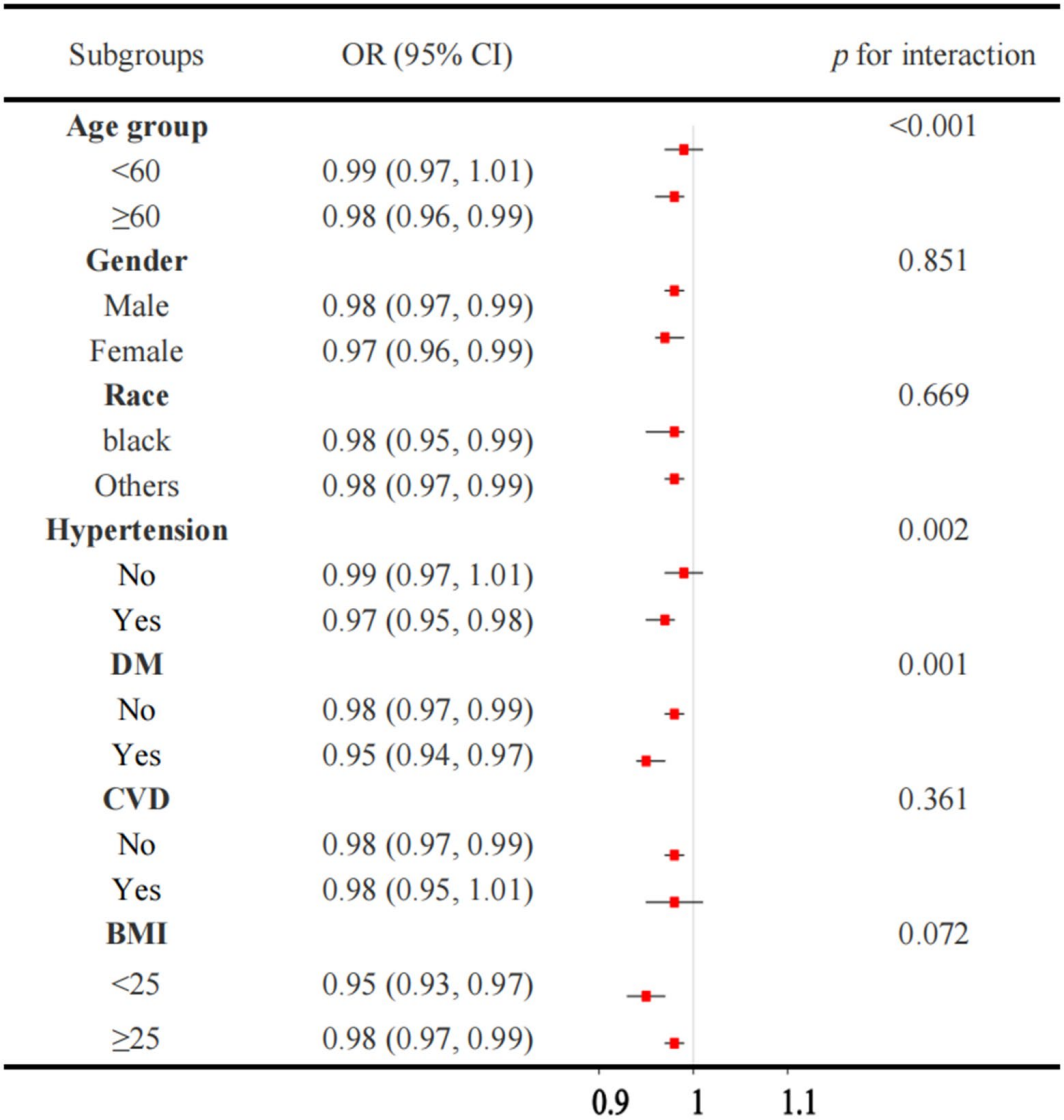
Association between the OBS and CKD in subgroup and interactive analyses. OBS, oxidative balance score. CKD, chronic kidney disease.

**Figure 4:**
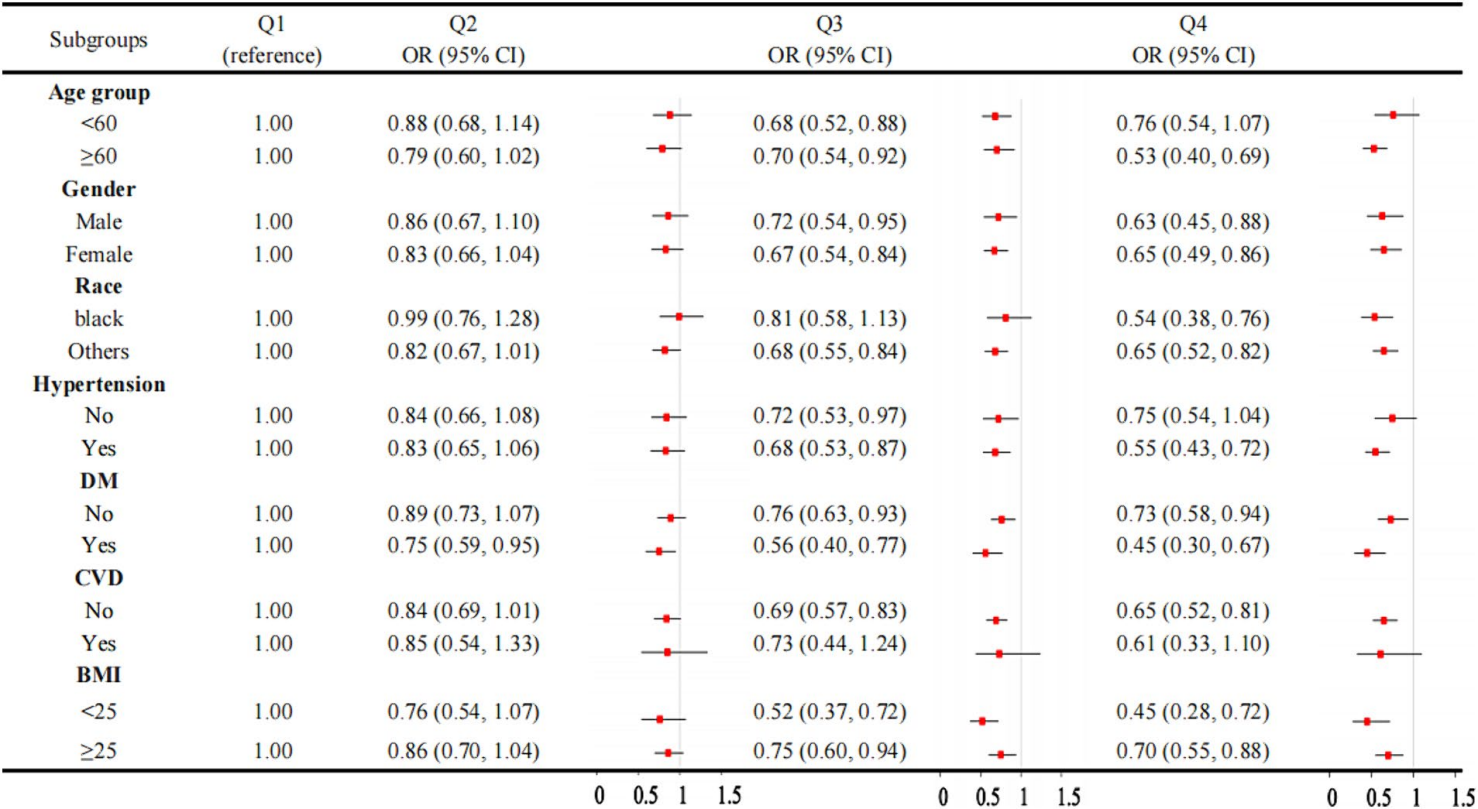
Association between OBS quartiles and CKD in subgroups. OBS, oxidative balance score. CKD, chronic kidney disease.

Figure 3 illustrated a robust association between OBS and CKD across the majority of the subgroups. However, the association is not significant in individuals without hypertension or those with CVD. When the OBS is divided into quartiles, the results are consistent, with the association between OBS and CKD remaining significant in most subgroups (Figure 4). However, this association is not statistically significant in Q4 compared to Q1 for individuals under 60 years of age, those without hypertension, and those with CVD. The interaction analysis reveals significant interactions between OBS and age, hypertension, and diabetes, indicating that these factors modify the effect of OBS on CKD risk.

## 4 Discussion

Drawing on data from NHANES 2009–2018, this research sheds light on the nexus between the OBS and CKD susceptibility. Our results, showcasing the protective influence of elevated OBS levels against CKD, were consistent with existing insights into oxidative stress’s contribution to CKD development. Oxidative stress has been identified as a significant contributor to the pathophysiology of CKD, particularly due to its role in promoting inflammation, vascular calcification, and proteinuria, all of which are risk factors for the progression of CKD in these patients. Oxidative stress is marked by a disequilibrium in oxidant production and the body’s antioxidative defense capacity. Prior research had highlighted that oxidative stress intensified as CKD advances, showing a strong association with the degree of kidney function (22–24). It appears that CKD is a pro-oxidant state, as evidenced by heightened levels of oxidative stress markers in clinical settings (such as elevated levels of plasma malondialdehyde, protein carbonyl, and F2-isoprostane) (7, 25–27). Indices related to metabolic dysfunction, such as TyG, have been associated with an increased risk of CKD, likely due to their connection to oxidative stress (11).

The inverse correlation between OBS levels and CKD risk suggests a protective role of a balanced oxidative profile, characterized by elevated antioxidant exposure and reduced pro-oxidant exposure. This relationship can be partly attributed to the reduction in oxidative stress and inflammation associated with higher antioxidant intake, which aligns with the pathophysiological processes involved in CKD progression. Antioxidants, such as those included in the OBS (e.g., vitamins C, E, *β*-carotene, selenium), work by neutralizing ROS and reactive nitrogen species, thereby reducing oxidative stress. By scavenging these reactive species, antioxidants can potentially mitigate the damage to cellular components, including lipids, proteins, and DNA, which is a key mechanism through which oxidative stress contributes to CKD progression. Additionally, previous studies have suggested that antioxidant therapy could have a protective effect against CKD progression by improving endothelial function and reducing inflammation. This is particularly relevant because patients with CKD are often in a state of heightened oxidative stress compared to the general population.

OBS included a broad range of 15 antioxidants such as dietary fiber, vitamins, and minerals, the exclusion of some plant bioactive compounds, specifically polyphenols, flavonoids, and other phytochemicals, from our OBS was due to several considerations. Firstly, although these plant bioactive compounds are recognized for their significant contributions to antioxidant activity, there are challenges associated with their inclusion in a standardized scoring system like the OBS. These challenges primarily stem from the diversity and complexity of these compounds, which have varying bioavailability and different effects depending on their specific types and interactions within the body. This makes it difficult to establish uniform measurement protocols and recommended intake levels for these compounds, unlike more established antioxidants like vitamins and minerals (18). Secondly, the OBS in our study was constructed using components that were consistently measured and reported across the dietary data of the study population. Components like vitamins C and E, selenium, zinc, and dietary fiber have well-documented antioxidant properties and are widely recognized and measured in nutritional studies, providing a more reliable basis for inclusion in the OBS. In contrast, the measurement of plant bioactive compounds like polyphenols and flavonoids is more variable, and thus their exclusion helps maintain the consistency and reliability of the OBS.

Several studies have highlighted the potential role of specific dietary antioxidants, such as *α*-Tocophero (28), zinc (29) and selenium (30), in mitigating the risk of CKD. However, findings have been inconsistent, with some studies reporting different outcomes. For instance, Park et al. found no significant association between dietary intake of vitamins A, C, and E and the progression of CKD to moderate or severe stages, even after adjusting for potential confounders (31). This suggests that simply increasing the intake of specific antioxidant vitamins May not be sufficient to impact CKD progression and underscores the need for a more integrated approach that considers the interplay of various dietary components and lifestyle factors in managing CKD. In our study, we employed the OBS as a practical and cost-effective method to assess the comprehensive balance of oxidative stress. Shi et al. reported that a higher Dietary Antioxidant Quality Score (DAQS), which focuses solely on dietary antioxidant intake, is correlated with a lower risk of hyperuricemia-related mortality among CKD patients (32). Li et al. also reported that dietary total antioxidant capacity, assessed using the Vitamin C Equivalent Antioxidant Capacity (VCEAC) and Component Dietary Antioxidant Index (CDAI), was associated with reduced all-cause mortality in early-stage CKD (stages 1–2) (33). These results support the idea that increased antioxidant intake is associated with better kidney function and reduced disease prevalence. However, these two indices only consider the role of dietary antioxidants in CKD, without accounting for the impact of lifestyle factors. Our study findings provide a valuable complement by incorporating both dietary and lifestyle influences.

This study also employed subgroup and interactive analyses to investigate potential interactive effects of covariates. Findings reveal notable interactive effects between the OBS and three conditions, age, hypertension and DM, individually. CKD, hypertension, and DM are all age-related disorders, with their prevalence escalating as individuals age. Oxidative stress has been identified as potentially responsible for the development of endothelial damage and vascular stiffness, both primary drivers in the development of hypertension (34, 35). Oxidative stress also plays a crucial and central role in the onset and progression of DM (36–38). A multitude of molecular events across various metabolic pathways, including glycolysis, hexosamine, protein kinase C, polyol, and advanced glycation end-products (AGEs), have been pinpointed as pro-oxidative mechanisms (36). These processes tend to be elevated in individuals with DM (36). Therefore, the observed interactions align with our expectations.

A key strength of our investigation was the deployment of an extensive, nationally representative cohort, enhancing the generalizability of our conclusions. Additionally, the detailed evaluation of the OBS, encompassing both dietary and lifestyle facets, offered a thorough appraisal of antioxidant and pro-oxidant exposures. Nonetheless, it was important to acknowledge that our study was subject to certain limitations. Firstly, we acknowledge the cross-sectional nature of the data, which limits our ability to establish causality between antioxidant intake, lifestyle factors, and CKD outcomes. Secondly, we recognize the reliance on self-reported information for dietary and lifestyle factors as another limitation. Self-reported data are subject to recall bias and inaccuracies, which could potentially affect the validity of our findings. To mitigate this, we have emphasized the need for future studies to employ longitudinal designs and utilize more objective measures of dietary intake and lifestyle factors to confirm our findings. Furthermore, the NHANES dataset predominantly includes individuals from the United States, and as such, our findings May be limited by the specific demographic and geographic context of this population. Factors such as diet, lifestyle, access to healthcare, and socioeconomic status can vary significantly between countries and regions, potentially influencing the applicability of our results to non-U.S. populations. Further studies are needed in diverse populations to fully understand the broader applicability of these findings.

## 5 Conclusion

To conclude, our investigation over five NHANES periods from 2009 to 2018 highlighted an inverse relationship between OBS and CKD among American adults. This association remained robust even after comprehensive control for potential confounding variables. Moving forward, employing randomized controlled trials or prospective cohort investigations holds promise in establishing the prognostic significance of the OBS within the CKD framework.

List of abbreviations.

albumin (ALB); body mass index (BMI); chronic kidney disease (CKD); cardiovascular disease (CVD); diabetes mellitus (DM); estimated glomerular filtration rate (eGFR); glomerular filtration rate (GFR); hemoglobin A1c (HbA1c); high-density lipoprotein (HDL); hemoglobin (HGB); the National Health and Nutrition Examination Survey (NHANES); Oxidative balance score (OBS); Oxidative stress(OS);Restricted cubic spline (RCS); serum creatinine (Scr); total cholesterol (TC); triglyceride (TG); urinary albumin/creatinine ratio (UACR).

## Data Availability

Publicly available datasets were analyzed in this study. This data can be found at: https://wwwn.cdc.gov/nchs/nhanes/.

## Footnotes

^1^https://www.cdc.gov/nchs/nhanes/

^2^https://www.R-project.org; R Foundation, Austria
